# Senescence of T cells and organ aging

**DOI:** 10.1186/s41232-026-00419-3

**Published:** 2026-04-24

**Authors:** Masaki Ohyagi, Minako Ito, Akihiko Yoshimura

**Affiliations:** 1 Division of Molecular Pathology, Research Institute for Biomedical Sciences, Tokyo University of Science, 2669 Yamazaki, Noda-City, Chiba 278-0022 Japan; 2 Department of Neurology and Neurological Science, Institute of Science Tokyo, 1-5-45 Yushima, Bunkyo-Ku, Tokyo, 113-8519 Japan; 3 Division of Allergy and Immunology, Medical Institute of Bioregulation, Kyushu University, Fukuoka, 812-8582 Japan

**Keywords:** Immunosenescence, Inflammaging, T cells, PD-1/PD-L1, Chemokine, NR4a, Spatial transcriptomics

## Abstract

Senescence of T cells is strongly linked to organismal aging through two interconnected processes: chronic low-grade inflammation and reduced immune surveillance of senescent cells. T cells are particularly vulnerable to thymic involution, hematopoietic stem cell aging, repeated homeostatic proliferation, chronic antigenic stimulation, and metabolic and mitochondrial dysfunction. As a result, aged T cells may lose their capacity to combat infection and eliminate senescent cells, while also contributing to inflammaging through the production of inflammatory cytokines. Recent preclinical studies in murine models have demonstrated that modulation of T-cell immunosenescence can ameliorate age-related diseases. These approaches include PD-1/PD-L1 blockade, senolytic chimeric antigen receptor T (CAR-T) cells, and CXCL4/platelet factor 4 (PF4). In addition, early-stage human clinical studies of caloric restriction, low-dose mTOR inhibition, thymic regeneration, and mesenchymal stromal/stem cell (MSC) therapy suggest that interventions targeting immunosenescence may provide health benefits. Moreover, in murine models of Alzheimer’s disease, T cells infiltrating the brain may exert either disease-promoting or protective effects depending on the disease stage, highlighting an important point of intersection between T-cell-mediated immunosenescence and brain aging. This review summarizes the basic concepts of immunosenescence, the molecular basis of immune surveillance of senescent cells, age-associated T-cell subsets, their links to brain aging, and interventional strategies aimed at clinical translation, with particular emphasis on T-cell biology and the transcriptional regulatory network driven by NR4a.

## Introduction

The age-associated increase in susceptibility to infection, malignancy, autoimmunity, neurodegeneration, and frailty cannot be explained simply by a decline in “immune strength.” Rather, aging entails a qualitative remodeling of the immune system. In the aged immune system, the capacity to eliminate pathogens and abnormal cells declines, while the tendency to generate persistent, low-grade inflammation increases. This duality is the essence of immunosenescence and provides a critical framework for understanding the progression of organismal aging.

**Fig. 1:**
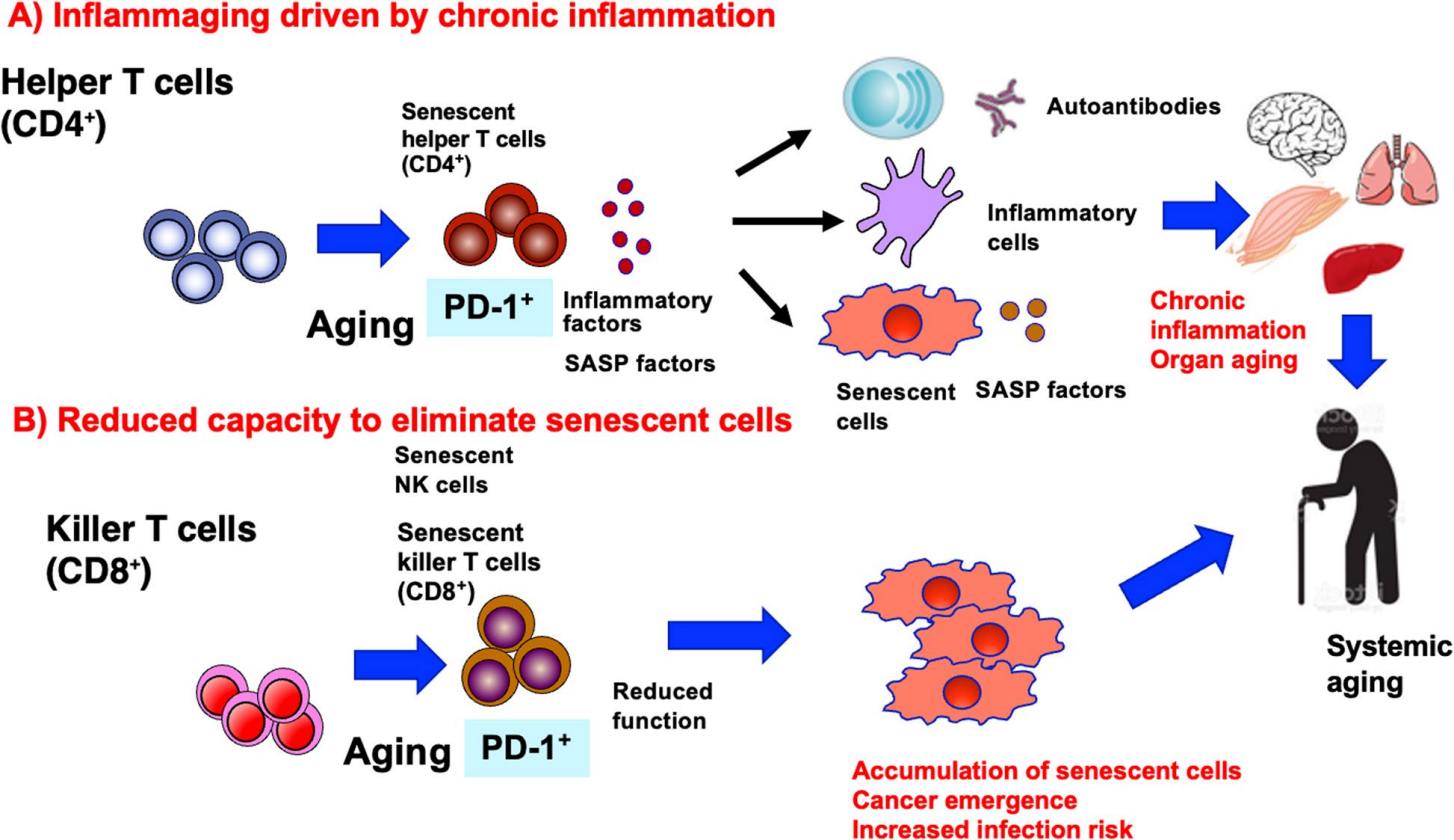
T-cell aging promotes organismal aging through two major mechanisms. **A** Senescent helper T cells (CD4^+^) and senescent macrophages increase the production of inflammatory cytokines. This promotes chronic inflammation, autoantibody production, and induction of SASP factors from senescent cells, thereby accelerating organ and systemic aging. **B** Senescence of NK cells and killer T cells (CD8^+^) causes functional decline, leading to cancer development, increased susceptibility to infection, and accumulation of senescent cells in tissues, which further accelerates tissue and organismal aging

Immunosenescence has two major faces. First, CD4^+^ T cells, macrophages, senescent cells, and other cell types continuously release inflammatory cytokines, chemokines, prostaglandins, and SASP (senescence-associated secretory phenotype) factors, thereby creating a state of chronic systemic inflammation. This chronic inflammation accelerates organ injury, autoantibody production, fibrosis, and neurological dysfunction, and thus directly contributes to aging phenotypes. Second, the ability of CD8^+^ T cells and NK cells to eliminate senescent, malignant, and infected cells declines. As a consequence, senescent cells accumulate, promoting tumorigenesis, vulnerability to infection, and further amplification of SASP-driven inflammation (Fig. 1).

Although immunosenescence involves multiple immune lineages, this review adopts a deliberately T-cell-focused perspective because T cells sit at the intersection of inflammaging, senescent-cell surveillance, thymic involution, and adaptive immune dysfunction. Where relevant, we also discuss macrophages, NK cells, and B cells to place T-cell aging within the broader immune landscape.

## Senescent CD4^+^ and CD8^+^ T cells

**Fig. 2:**
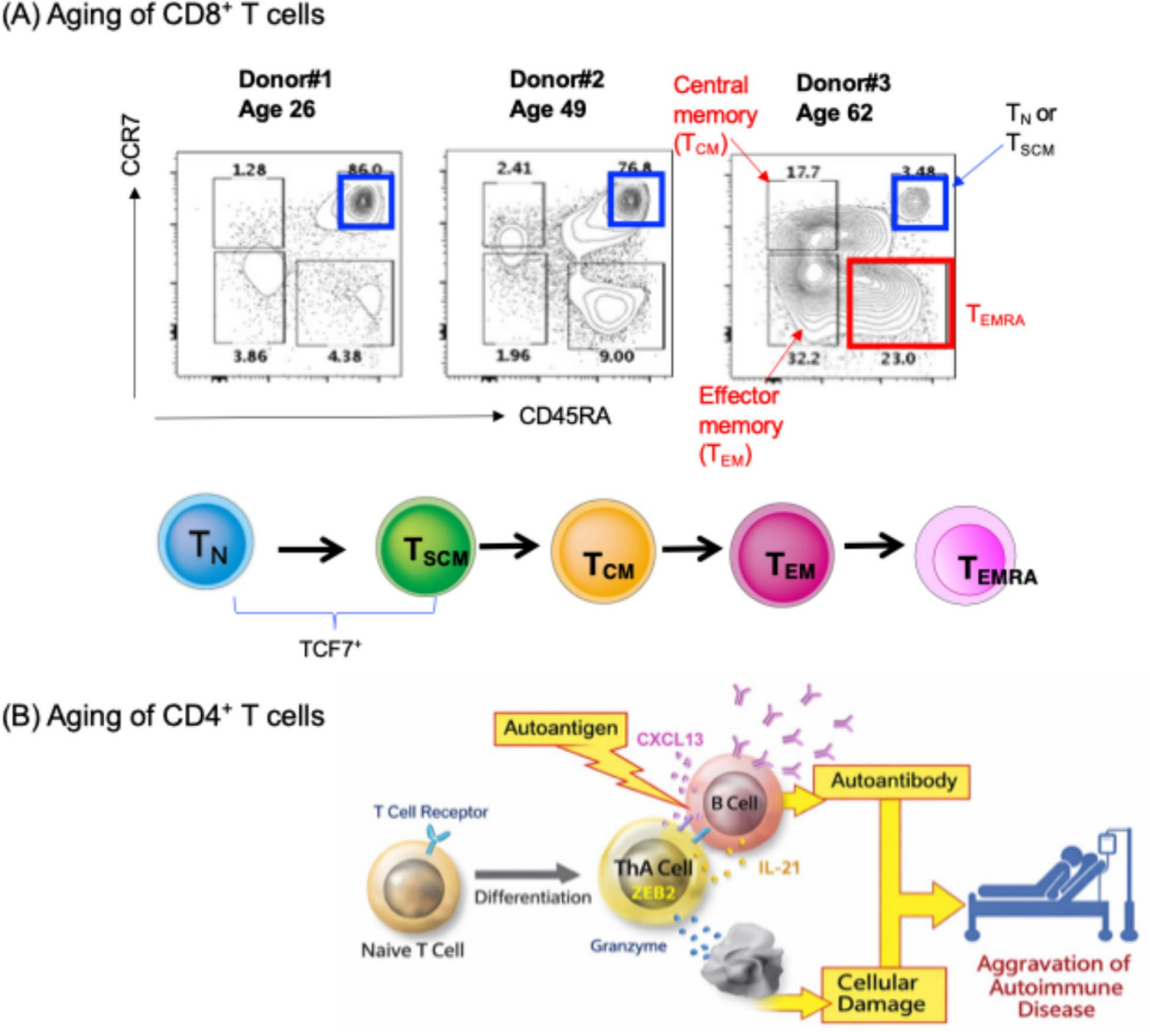
Age-associated differentiation of human CD8^+^ and CD4^+^ T cells. **A** Aging of CD8^+^ T cells. Representative FACS profiles of CD8^+^ T cells from three donors of different ages based on the differentiation markers CCR7 and CD45RA. After 60 years of age, the CCR7⁺CD45RA⁺ naïve (T_N_) or stem cell memory (T_SCM_) fraction markedly decreases, whereas populations including terminally differentiated effector memory T cells re-expressing CD45RA (T_EMRA_), which display an exhausted phenotype, increase. Adapted from Ando et al. [1]. The lower scheme shows the order of differentiation. T_N_ and T_SCM_ cells express high levels of TCF7. **B** Aging of CD4^+^ T cells. Fujio and colleagues at the University of Tokyo described these populations as age-associated CD4^+^ T-cell subsets (ThA cells), in which the transcription factor ZEB2 plays an important role [4]. These cells are characterized by increased IFN production and acquisition of cytotoxicity. They can promote autoantibody production by B cells and contribute to autoimmunity, while their cytotoxic activity may also contribute to tissue injury and to the elimination of senescent cells

At least two upstream processes underlie T-cell aging. The first is aging of hematopoietic stem cells, which reduces lymphoid output and relatively favors myeloid differentiation. The second is thymic involution, which markedly decreases the generation of new naïve T cells with age. In the periphery, these changes force repeated homeostatic proliferation of preexisting T cells, leading to contraction of the TCR repertoire, relative enrichment of self-reactive clones, and accumulation of terminally differentiated or exhaustion-like phenotypes. In humans, naïve T cells and stem cell memory T (T_SCM_) cells decline after approximately 60 years of age, whereas terminally differentiated CD8^+^ T cells, including terminally differentiated effector memory T cells re-expressing CD45RA (T_EMRA_) cells, increase [1] (Fig. 2A).

Within the CD4 compartment, the diversity of age-associated T-cell states is also interesting. Tertiary lymphoid tissues that develop in aged injured kidneys in mice and humans accumulate PD-1⁺CD153⁺ senescence-associated T (SAT) cells that produce osteopontin, IL-6, IFN-γ, and related inflammatory mediators, thereby sustaining chronic inflammation [2]. By contrast, human supercentenarians exhibit an expansion of granzyme B-positive cytotoxic CD4^+^ T cells [3], and in autoimmunity of human, ZEB2-dependent age-associated helper T (ThA) cells have been shown to combine B-cell help with cytotoxic activity [4]. Thus, aged CD4^+^ T cells can share seemingly opposing functions: promotion of autoimmunity and elimination of senescent cells (Fig. 2B).

Accordingly, a key goal in anti-aging immunology is to restore immune balance—suppressing excessive inflammation while preserving host defense, vaccine responsiveness, and immune surveillance against senescent cells.

## Properties of senescent cells and their immunological elimination

A central trigger of cellular senescence is DNA damage, which can be induced by replication stress, telomere shortening, oxidative stress, mitochondrial dysfunction, and inflammatory reactive oxygen species. DNA damage responses activate p53, p16, and p21, leading to cell-cycle arrest. Senescent cells thereby lose proliferative capacity, yet actively remodel the surrounding tissue and immune microenvironment through secretion of SASP factors. Although the SASP can contribute to local repair in some contexts, chronic SASP signaling promotes inflammation, fibrosis, stem-cell dysfunction, and secondary senescence in neighboring cells, thus establishing a self-amplifying circuit of aging.

In young individuals, many of these senescent cells are removed by immune surveillance. With aging, however, senescent cells themselves acquire immune-evasion mechanisms. Wang and colleagues showed that a subset of p16-positive senescent cells highly expresses PD-L1 and thereby escapes T-cell-mediated killing in mice. PD-1 antibody treatment improved senescent-cell clearance and attenuated selected aging phenotypes such as fatty liver and decreased grip strength, suggesting that failure of senescence surveillance can involve immune-checkpoint pathways [5].

The significance of the PD-1/PD-L1 axis is also under discussion in neurodegenerative disease. In murine models of Alzheimer’s disease (AD) and tauopathy, PD-1 blockade has been reported to recruit monocyte-derived macrophages and improve pathology [6, 7], although other studies failed to reproduce such benefits, indicating substantial dependence on timing, dosing, and disease model [8].

Classically, CD8^+^ T cells and NK cells have been regarded as the major effector populations responsible for eliminating senescent cells as shown in Fig. 1B. More recently, however, cytotoxic CD4^+^ T cells (CD4-CTLs) in human skin have emerged as important effectors in this process. Hasegawa et al. demonstrated that senescent fibroblasts present HLA class II and CMV antigens, which are recognized by resident CD4-CTLs that then kill these senescent cells [9]. In mice, Eomes-positive CD4^+^ T cells also arise with age, and loss of this population leads to increased senescent-cell accumulation, reduced physical function, and increased mortality [10]. Thus, the CD4 lineage has a dual nature in both humans and mice: it can exacerbate autoimmunity and tissue damage, yet also contribute to clearance of senescent cells.

In parallel with pharmacological senolytics, strategies that confer senescent-cell specificity directly on immune cells have attracted considerable attention. Amor et al. showed that CAR-T cells targeting uPAR, a molecule expressed on senescent cells, can ameliorate senescence-associated pathologies in mice [11, 12]. This approach most directly embodies the concept of “using immunity to clean up senescent cells.”

## T-cell aging as a potential contributor to organismal aging

A compelling demonstration in a murine model showed that T-cell immunosenescence can, in at least some experimental settings, contribute directly to organismal aging. Desdín-Micó and colleagues showed that T-cell-specific deletion of the mitochondrial transcription factor TFAM in mice induces lysosomal abnormalities, a reduced NAD^+^/NADH ratio, impaired proliferation, and increased TNF-α and IFN-γ production in T cells, culminating in systemic accumulation of senescent cells, multi-organ dysfunction, physical decline, and premature death [13]. Importantly, these phenotypes were partially rescued by anti-TNF-α antibody treatment or by administration of NAD precursors. These findings experimentally support a model in which metabolic and mitochondrial dysfunction in T cells drives systemic aging through chronic inflammation.

Is there a correlation between T-cell immunosenescence and healthy lifespan in humans? As mentioned earlier, T-cell aging is driven primarily by thymic involution. Researchers at Harvard University led by Scadden have reported epidemiological evidence that patients who underwent thymectomy for various reasons show reduced T-cell replenishment and increased mortality [14]. More recently, a cohort study using a CT-based method for assessing the thymus demonstrated that thymic health remains associated with mortality and several metabolic and inflammatory parameters in adults [15]. The same group simultaneously published another paper examining the relationship between this thymic health index and responses to immune checkpoint inhibitor (ICI) therapy in patients with cancer [16]. In that study, better thymic health was associated with markers consistent with de novo T-cell production and with improved ICI responses in cancer types that are generally more responsive to ICI therapy, such as lung cancer and melanoma.

Taken together, these findings suggest that thymic health may reflect, at least in part, the capacity to generate new T cells in adults. Even so, the currently available human data are largely observational or linked to cancer immunotherapy settings, and therefore should not yet be interpreted as proving a broad causal role of the thymus in age-related disease or aging overall.

## Spatial organization of immune aging in tissues

Immunosenescence is not only a systemic process but also a spatially organized one. Age-associated immune remodeling unfolds within distinct tissue niches, including tertiary lymphoid structures, mucosal sites, lymphoid organs, and the aging brain, where local stromal cues, vascular barriers, resident myeloid cells, and tissue architecture collectively shape immune-cell behavior. Recent advances in spatial transcriptomics, multimodal tissue atlases, and imaging-based approaches are beginning to reveal how inflammatory programs, immune-cell localization, and cell–cell interactions are arranged within aging tissues [17–19]. Importantly, these studies indicate that age-associated immune remodeling is often concentrated in discrete anatomical niches rather than being uniformly distributed across organs. For example, spatial transcriptomics in the aging mouse brain identified white matter fiber tracts as a focal site of inflammation, characterized by microglial activation, complement-associated programs, and myelin loss [17]. Complementing this, multimodal profiling in human blood and lymphoid and mucosal tissues demonstrated that tissue-level immunosenescence is strongly tissue-directed, with prominent age-associated alterations in mucosal macrophages, lymphoid-organ B cells, and circulating T and NK cells across tissues [18]. In addition, imaging-based spatial analyses using multiplexed error-robust fluorescence in situ hybridization (MERFISH) have begun to show that cell-proximity relationships themselves influence aging-associated states in the brain, underscoring the importance of local cellular neighborhoods in tissue aging [19].

This spatial perspective is highly relevant to the broader concept of immunosenescence, even if spatially resolved evidence for T-cell aging per se remains more limited than that for tissue-level immune remodeling. Nevertheless, it is also pertinent to T-cell biology, because the consequences of T-cell infiltration depend not only on lineage and differentiation state but also on where these cells accumulate and which local circuits they engage. The brain provides a useful example: within this highly specialized microenvironment, blood-borne rejuvenating factors and brain-infiltrating T cells appear to exert context-dependent effects that are shaped by local myeloid cells, barrier structures, and tissue architecture.

## “Rejuvenation” of the brain and immune system by chemokines

The idea that blood-borne factors from young organisms can improve function in aged organisms emerged from studies of heterochronic parabiosis. CCL11 (eotaxin) was identified as a pro-aging factor in the serum of aged mice. Although CCL11 is well known as a chemokine involved in allergic responses and in the recruitment of Th2 cells and eosinophils, the precise mechanisms by which it promotes neural aging remain incompletely understood [20]. Villeda et al. subsequently showed that young blood can improve cognitive function and synaptic plasticity in aged mice [21]. Thereafter, several groups identified the platelet-derived chemokine PF4/CXCL4 as a youthful factor capable of improving cognition, neurogenesis, and immune profiles in aged mice [22–24]. In humans, CCL11 levels increase in plasma and cerebrospinal fluid with age, whereas PF4/CXCL4 levels decline.

However, the mechanisms by which PF4/CXCL4 improves brain function remain unclear. Its beneficial effects may be mediated not only through direct actions on the brain but also through modulation of peripheral immune states. Among the three groups that identified PF4/CXCL4 as a brain “rejuvenation” factor, Schroer et al. focused on the immune system because CXCR3, a receptor for PF4, is highly expressed on T cells but not on neural cells [22]. They showed that CXCL4 administration suppresses inflammatory signaling that is increased with age, corrects the myeloid-to-lymphoid imbalance, and reduces T-cell aging markers in aged animals. Brain-associated T cells may therefore not only exacerbate neurodegeneration but also participate in the clearance of degenerating cells or facilitate amyloid-β clearance. From the perspective of the intersection between immunosenescence and brain aging, the CXCL4–T-cell–brain axis represents an important subject for future investigation.

## T cells and immunosenescence in Alzheimer’s disease

Alzheimer’s disease (AD) is a prototypic neurodegenerative disorder characterized by the accumulation of amyloid-β (Aβ) and tau. Recent studies in patients with AD have demonstrated the presence of clonally expanded T cells, particularly CD8^+^ T cells, in the brain and cerebrospinal fluid, renewing interest in the role of adaptive immunity in neurodegeneration [25]. However, whether brain-infiltrating T cells aggravate pathology or exert protective functions remains unresolved.

In murine AD models, such as AppNL-G-F and 5xFAD mice, the number of brain CD8^+^ T cells increases as amyloid pathology progresses with age. However, the role of brain CD8^+^ T cells in these models has been controversial [26, 27]. We recently demonstrated that the effects of CD8^+^ T cells on AD pathology are time dependent [28]. T-cell deficiency or CD8 depletion reduces Aβ deposition during the early phase of disease but increases Aβ deposition at later stages, indicating that CD8^+^ T cells may act in a pathogenic manner early in the disease course but become suppressive or protective at later stages in these models [28].

**Fig. 3:**
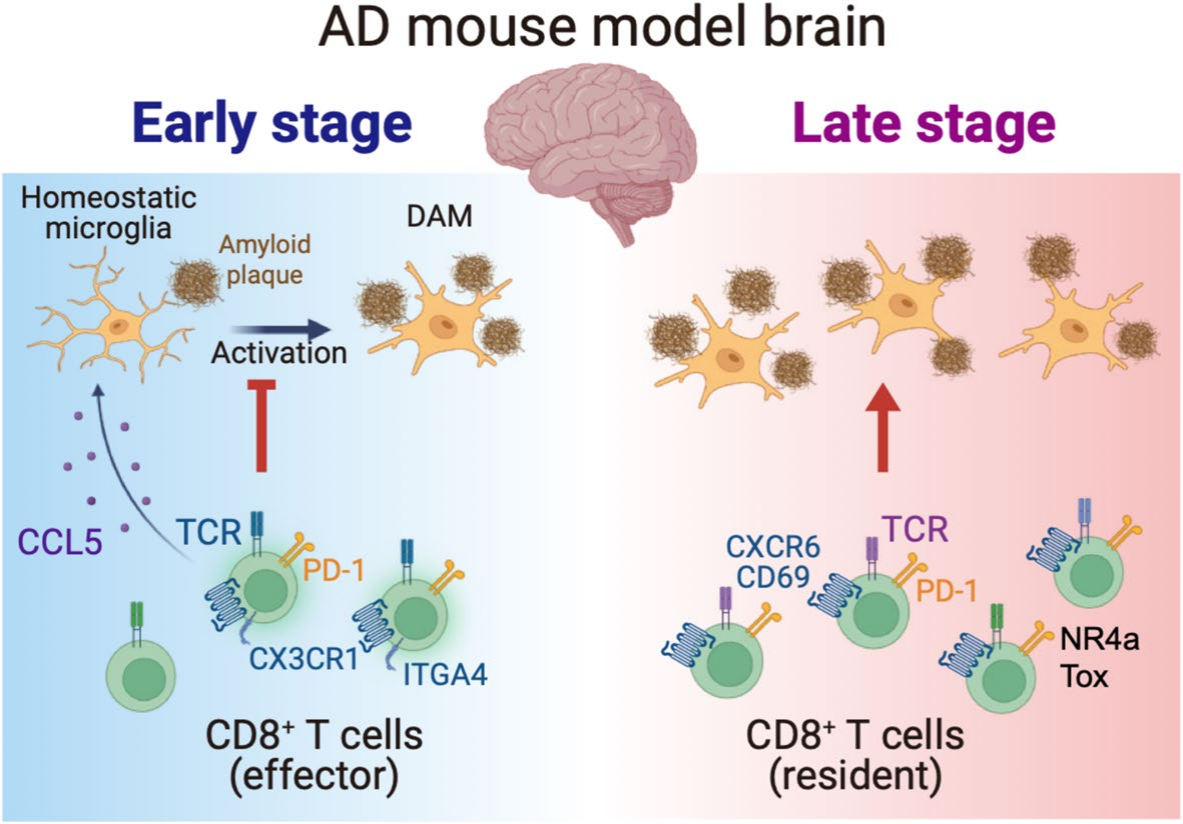
Stage-specific roles of CD8^+^ T cells in amyloid pathology. In murine AD models, CD8^+^ T cells can promote pathology during the early phase of amyloid deposition but may exert suppressive or protective effects at later stages. Detailed analysis of CD8^+^ T cells infiltrating the brain early in disease showed that these cells had already undergone clonal expansion and highly expressed molecules involved in activation and brain infiltration, including PD-1, CX3CR1, and CD49d. The CCL5-CCR5 pathway was activated: CD8^+^ T cells produced CCL5, whereas microglia expressed CCR5. During the progressive phase after 8 months of age, CD8^+^ T cells changed their phenotype and expressed tissue-residency markers such as CD69 and CXCR6, as well as exhaustion-associated markers including NR4a and TOX. These T cells may instead enhance the amyloid-reactive state of microglia and function protectively against amyloid pathology at later stages

CD8^+^ T cells infiltrating the brain at early stages undergo clonal expansion and highly express activation- and brain-homing-associated molecules such as PD-1, CX3CR1, and CD49d. Moreover, CD8^+^ T cells engineered to express T-cell receptors (TCRs) reactive to antigens derived from amyloid-laden brains selectively infiltrate the brain and exacerbate amyloid pathology, suggesting that antigen-specific CD8^+^ T cells can promote pathology in early lesions. One possible mechanism is that CCL5 derived from CD8^+^ T cells alters pathological microglial responses and suppresses differentiation into disease-associated microglia (DAM), which are capable of Aβ phagocytosis [28] (Fig. 3).

By contrast, CD8^+^ T cells present at advanced stages express markers associated with tissue residency or exhaustion, including CD69, CXCR6, NR4a, and TOX, and may instead promote amyloid-reactive microglial responses that help restrain pathology [28]. Thus, T cells in the AD brain should not be viewed as uniformly pathogenic. Rather, the available data suggest that they can exert opposing functions depending on disease stage, differentiation state, and antigen specificity. In the context of immunosenescence, exhaustion and terminal differentiation of brain-infiltrating T cells may not simply reflect loss of function, but may also constitute a stage-specific regulatory mechanism in AD pathogenesis. Clarifying this issue will be an important task for future studies.

## NR4a, a transcription factor that controls expression of immune checkpoint molecules

Aged and exhausted T cells coexpress multiple inhibitory receptors, including PD-1, TIM-3, LAG-3, and TIGIT, together with signaling suppressors such as SOCS family members. In cancer immunology, these molecules are collectively referred to as immune checkpoint molecules. Chromatin regions specifically accessible in exhausted T cells are enriched for binding motifs of NFAT and the NR4a family [29–32], and exhaustion-associated enhancers upstream of the Pdcd1 locus contain binding sites for both NR4a and NFAT [32, 33].

**Fig. 4:**
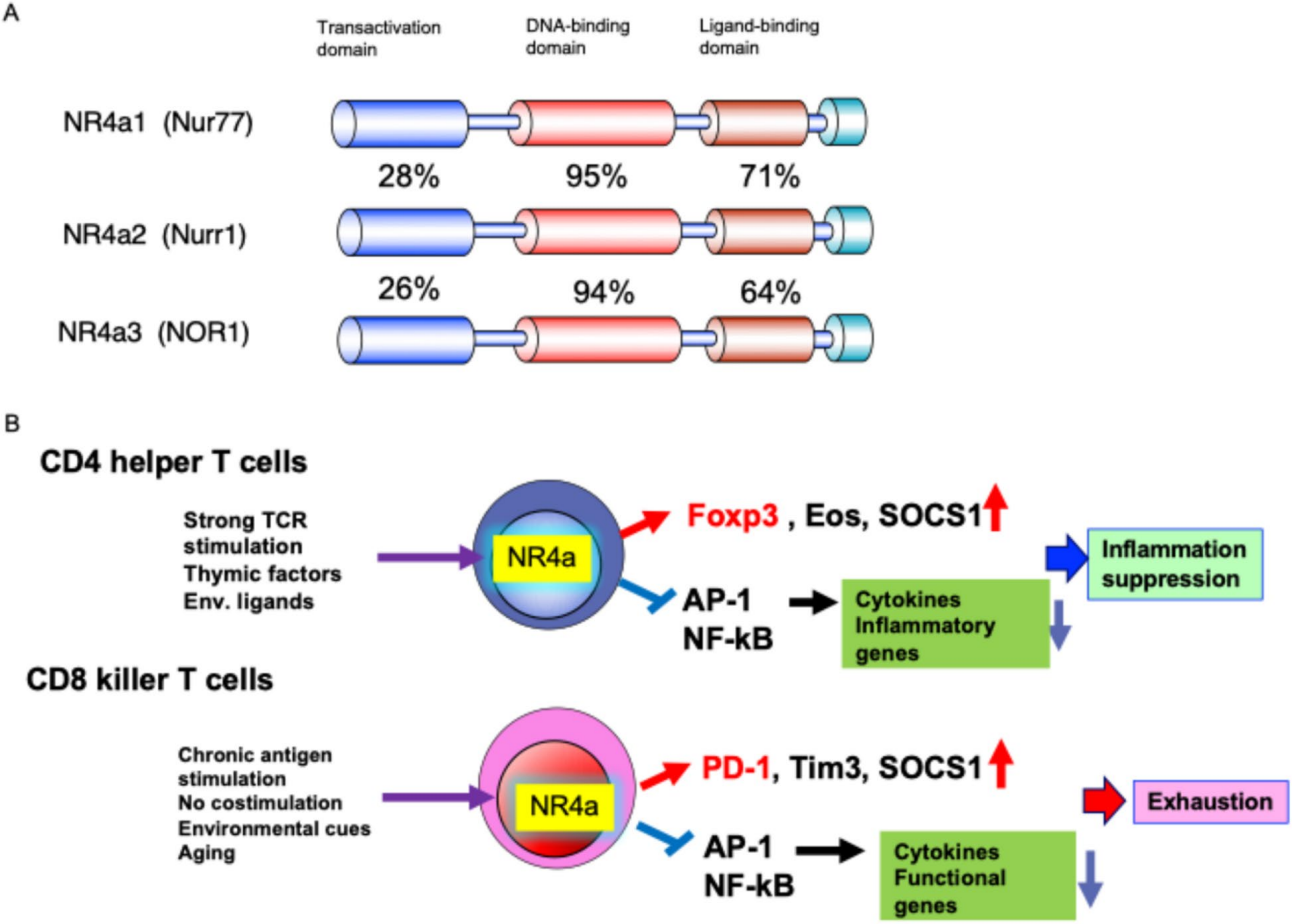
Structure of NR4a and its functions in T cells. **A** Comparison of the structures of the three NR4a family members. Numbers indicate the degree of homology to NR4a1 in each domain. Although the transcriptional activation domains are less similar, the DNA-binding domain, ligand-binding domain, and C-terminal region are highly conserved. **B** Functions of NR4a in CD4^+^T cells and CD8^+^ T cells. NR4a is induced in CD4^+^T cells by strong TCR stimulation and in CD8^+^ T cells by chronic antigen stimulation. It directly enhances the expression of Foxp3, Eos (Ikzf4), PD-1, and Tim3, while suppressing cytokine and cytotoxic effector gene expression through regulation of NF-kB and AP-1

The NR4a family comprises three orphan nuclear receptor transcription factors—NR4a1 (Nur77), NR4a2 (Nurr1), and NR4a3 (Nor1)—that are broadly conserved from nematodes to mammals and govern diverse processes including metabolic regulation, lifespan, and cell differentiation [34]. We previously identified NR4a as an essential transcription factor for the development and maintenance of regulatory T (Treg) cells, showing that it promotes expression of Foxp3 and Ikzf4 (Eos) while suppressing expression of cytokine genes such as IL-4, IL-21, and IFN-γ [35–37] (Fig. 4).

In conventional T cells, NR4a is transiently induced by TCR stimulation. By contrast, under chronic antigen stimulation in exhausted T cells, or in anergic T cells receiving TCR signals in the absence of costimulation, NR4a expression remains persistently elevated [38, 39]. In human tumor-infiltrating lymphocytes, NR4a is likewise highly expressed in exhausted T cells. NR4a directly binds inhibitory receptor genes such as PD-1 and TIM-3 to promote chromatin accessibility, while suppressing cytokine-gene expression by competing with NF-kB and AP-1 [38–40] (Fig. 4).

On the basis of these findings, inhibition of NR4a has been explored as a strategy to enhance antitumor immunity. In murine tumor models with CD8^+^ T-cell-specific deletion of NR4a1/2, exhausted tumor-infiltrating T cells are reduced and potent antitumor effects are observed [41]. Single-cell RNA-seq further showed that NR4a1/2 deletion not only diminishes exhausted populations but also increases TCF1-positive stem-like precursor cells [41]. In murine tumor models, these findings suggest that NR4a inhibition can, in some settings, shift exhausted T-cell states toward a less terminally differentiated and more progenitor-like phenotype, rather than definitively “rejuvenating” them.

## NR4a in aging and immunosenescence

Although the role of NR4a in T-cell exhaustion is now well established, its direct relationship to T-cell aging and organismal aging remains incompletely defined. In a murine high-fat-diet-induced metabolic dysfunction-associated steatotic liver disease (MASLD) model, liver fibrosis is suppressed in mice lacking NR4a1/2 specifically in T cells [42]. Conversely, activation of NR4a may increase the abundance of Treg cells and restrain chronic inflammation. Indeed, the bile acid metabolite isoalloLCA, identified as a characteristic metabolite of the gut microbiota of human centenarians [43], increases Treg cells and suppresses inflammatory Th17 cells in mice [44], and part of this effect has been reported to be mediated through NR4a1 [45]. Resveratrol, which has long been considered beneficial for human health, has been shown to act as an NR4a1 antagonist [46], whereas inhibition of NHR-6, the *C. elegans* ortholog of NR4a, shortens lifespan [47]. Taken together, these findings suggest that NR4a signaling participates in the regulation of inflammation, metabolism, and longevity in a context-dependent manner.

Recent studies have also revealed a role for NR4a in B-cell aging in mice. NR4a1/2/3 are rapidly induced by B-cell receptor stimulation and restrain B-cell responses by suppressing BATF and MYC when T-cell help is absent or limited [48]. Conversely, in self-reactive anergic B cells, reduced NR4a1 expression induced by T-cell-derived cytokines or TLR7/9 signaling can lead to reactivation, differentiation into age-associated B cells (ABCs), and autoantibody production [49]. NR4a may thus function as a hub linking immunosenescence across T cells, Treg cells, and B cells.

## Attempts to target immunosenescence in humans

Efforts to modulate immunosenescence in humans now span lifestyle interventions, pharmacological approaches, and cell-based therapies. Two years of moderate caloric restriction was associated with reduced thymic fat deposition, improved thymic output, and lower levels of inflammation [50]. Low-dose TORC1 inhibition using a combination of RAD001 and BEZ235 reduced infection rates and enhanced vaccine responses in older adults, suggesting that this approach acts not as broad immunosuppression but rather as selective immune recalibration [51].

A thymic regeneration trial combining growth hormone, dehydroepiandrosterone (DHEA), and metformin reported increased thymic mass, an improved lymphocyte-to-granulocyte ratio, fewer PD-1-positive T cells, and a reduction in epigenetic age [52]. However, this was a small single-arm study, and its reproducibility and long-term safety remain to be established. Urolithin A, a mitophagy inducer, has been shown to increase naïve-like T cells and alter inflammatory and metabolic transcriptional programs in a randomized controlled trial, highlighting its potential as an adjunctive intervention for immunosenescence [53]. In addition, the mesenchymal stromal/stem cell (MSC) product laromestrocel, which has been approved by the FDA, has been reported to attenuate brain atrophy and cognitive decline in mild Alzheimer’s disease, an effect that may partly reflect the correction of chronic inflammation [54].

Nevertheless, improvements in immune parameters cannot yet be assumed to translate directly into an extension of healthy lifespan. Future studies will need to integrate multiple clinical outcomes, including host defense, vaccine responsiveness, frailty, cognition, cancer incidence, and autoimmunity, and determine which interventions are most appropriate for specific patient populations.

## Concluding remarks

Senescence of T cells, chronic inflammation, accumulation of senescent cells, and decline in organ function are tightly interconnected and together shape systemic aging. In humans, observational and atlas-level studies indicate that aging is associated with reduced thymic health, remodeling of tissue immune compartments, accumulation of age-associated T-cell states, and diminished functional reserve in settings such as infection, vaccination, and cancer immunotherapy. These findings strongly support clinical relevance, although they do not by themselves establish causality.

In murine models, causal relationships can be tested more directly. Experimental studies show that T-cell mitochondrial dysfunction can drive systemic senescence phenotypes, that brain-infiltrating CD8^+^ T cells can exert stage-dependent effects in Alzheimer-like pathology, and that interventions targeting the PD-1/PD-L1 axis, NR4a-dependent transcriptional programs, senescent cells, or youthful circulating factors such as PF4/CXCL4 can modify age-associated phenotypes.

Taken together, evidence from human studies, murine models, and early clinical trials supports several general conclusions. First, in humans, immune aging is consistently associated with thymic involution, altered tissue immune composition, and the accumulation of dysfunctional or age-associated lymphocyte states, but the causal hierarchy among these changes remains incompletely defined. Second, in mice, experimental manipulation has demonstrated that T cells are not merely biomarkers of aging but active drivers or regulators of age-related pathology, depending on tissue context and disease stage. Third, across both humans and murine models, the findings most strongly supported are that immune aging is not a uniform decline but a context-dependent remodeling process, that T cells are central but not solitary regulators of this process, and that successful intervention will require recalibration rather than indiscriminate immune activation. Achieving clinical translation will therefore require an integrated perspective bridging basic immunology, aging research, neuroimmunology, and carefully designed interventional studies.

## Data Availability

No datasets were generated or analysed during the current study.
